# Translating evidence into policy: opinions and insights of Health Researchers and Policymakers in Nepal

**DOI:** 10.1186/s12913-021-07102-y

**Published:** 2021-10-09

**Authors:** Biplav Babu Tiwari, Anusha Ban, Sony Gurung, Khem Bahadur Karki

**Affiliations:** 1 Maharajgunj Medical Campus (MMC), Institute of Medicine (IOM), Tribhuvan University (TU), Kathmandu, Nepal; 2 College of Public Health, University of Georgia, Athens, Georgia; 3 College for Public Health and Social Justice, Saint Louis University, Saint Louis, USA

**Keywords:** Evidence-based policy, Health policy, Nepal, Research-to-policy, Qualitative research

## Abstract

**Background:**

The Evidence-Based Policymaking (EBP) process in Nepal is rife with poor practices where often policymakers are portrayed as perpetrators for such practices. However, we need to think of the EBP as a two-sided coin where both research pull and research push play equally significant roles. This study aimed to assess the perception of Nepalese health policymakers and researchers on EBP and identify appropriate mechanisms to integrate evidence into policies.

**Methods:**

Following the constructivist philosophical paradigm, qualitative research design was used in the study with the grounded theory approach. Purposive sampling was performed, and the 12 in-depth interviews were conducted, where number of interviews was finalized using saturation theory. All interviews were audio-recorded, transcribed, translated to English, coded line by line, and then developed into themes. Thematic analysis technique was used to manually analyze the data.

**Results:**

Study participants highlighted that evidence is being utilized during policy formulation but not in the amount it should be, with a preference for anecdotal evidence further reducing the chance. Apart from these barriers, poor credibility of information obtained, poorly targeted dissemination, inadequate policy-based researches, and policymakers and researchers operating within the spheres of their own with a feeble link to channel the flow of information between them were identified by participants. On the other hand, the publication of one-pager research brief, conduction of nationally representative surveys especially quantitative studies, the practice of cost-effectiveness study, and policymaker’s involvement during the research were some facilitators identified.

**Conclusions:**

Moreover, the study accentuates that better communication strategies such as the establishment of formal forums with policymakers and researchers, better-targeted dissemination, and identification of priority areas have wide potential to promote a unified front of health policymakers and researchers for EBP.

## Background

The evolution of utilization of evidence in the policymaking process for any country usually follows a spectrum starting from evidence-free policy (EFP) to policy-based evidence (PBE) and ultimately to evidence-based policymaking (EBP) [1]. EBP is an approach to help policymakers make a well-informed decision about policies, programs, and projects by putting the best available information at the heart of policy development to avoid or minimize policy failures caused by a mismatch between government expectations and actual, on-the-ground conditions [2, 3].

The need for EBP is well established in the global policy environment and the context of Nepal is no different [4]. Even though the need has been fully realized, the actions are low to nil. The National Health Policy 2019 has mentioned the importance of the use of health research for policy development [5], but the progress is poor and the factors for such delay can be attributed to both researchers and policymakers.[4] But much of the global attention on research to policy translation is focused on policymakers only [6, 7]. This focus is justifiable because they do play a major role but we usually tend to forget the other side of the coin i.e. health researchers and their role for research push [8]. The barriers for research to policy spectrum are from both policymakers and researchers [9], and studying them separately will further intensify the existing gap between them. Both the policymakers and researchers are responsible for creating barriers of their own. The barriers created by policymakers are political use of evidence, low budget allocations and lack of technical competency among policymakers whereas researchers create barriers such as low availability of priority-based researches, use of jargons, and poor dissemination activities [3, 4, 6–11]. These barriers lead to the formation of the evidence-free policy that does not address the population’s real needs and ultimately results in policy failures [2, 12, 13].

Thus, the study assesses perspectives and attitudes of Nepalese health policymakers and researchers together, regarding the use and impact of evidence in policymaking. Additionally, not only does the research help to identify key barriers and facilitators of EBP but also points out the appropriate mechanisms to integrate research evidence into policymaking.

## Methods

This study took a participatory constructivist philosophical paradigm to seek the understanding and actions of study participants. Following this paradigm, the qualitative research design was used to explore the perception and attitudes of participants on EBP. Further, aspects of grounded theory within qualitative design helped in the systematic generation of theory from a diverse array of information obtained from interviews.

The participants of this study were Nepalese health policymakers and researchers. Health policymakers were defined as government employees in the capacity of a section or division chief (9th level officer and above), working currently or in the past, at the Ministry of Health and Population (MoHP) and its departments. This also included health policy advisors from organizations other than MoHP that advocate for policy need and those who prepare briefing notes and policy documents that in turn shape policy determination. Health researchers were defined as individuals working as institutional and/or independent researchers conducting researches related to health and health system.

Purposive sampling of both health researchers and policymakers was done to identify the study population. The possible health researchers were identified by visiting health research institutes operating in Kathmandu while possible health policymakers were identified by visiting MoHP and Department of Health Services (DoHS). The possible participants were first approached with a proposal brief of the study to clarify study objectives and get further information on their past policy involvement. Afterwards, principal investigator prepared a prioritized list of participants based on their past policy involvement, free schedule, and willingness to participate in the study. All participants were approached, following the prepared prioritized list, accepted to participate in the research interview. The total number of participants was finalized following the saturation theory which included 5 health policymakers and 7 health researchers.

Before all interviews, consent was obtained from participants following a verbal explanation and upon reading and signing an informed consent form. Face-to-face, semi-structured, in-depth interview was conducted with all 12 key informants at their workplace in a separate room with the presence of only researcher and participant. Two interview guides were developed, each for health policymakers and researchers, based on similar studies conducted in Argentina [6, 7] and were adopted in the context of Nepal to explore maximum information from participants (the interview guide is provided as additional file titled “ Interview Guidelines”). The interview was conducted by a single person i.e. corresponding author (male) who at the time was a 4th year Bachelor of Public Health (BPH) student with academic training on qualitative research. All 12 interviews were one-on-one, conducted by a principal investigator only, which lasted between 30 and 45 min. The interviews were conducted during September and October of 2018. Detailed field notes were taken during the interviews and audios were recorded after receiving written and verbal consent from the participant.

All the authors were involved during the data analysis and coding process. Interviews were then transcribed in the Nepali language except for one interview which was conducted in English. The interview in English was conducted with participant R06, where the participant preferred English as a form of communication during the interview. The Nepali transcripts were translated into English before further processing the data. The Three-step coding approach by Strauss and Corbin was used during the coding process i.e. open coding, axial coding, and selective coding.[14] Before it, in-vivo codes were generated through the process of the line by line codes. These in-vivo codes were grouped in terms of their similarity and then subjected to open coding. Data were reduced and narrowed to certain themes based on the inductive approach. Inductive approach helped to identify relationship among the information generated through interviews and form theories based on them. Open codes in Microsoft Word were exported to Microsoft Excel. Codes with similar meanings were put together and organized as a theme into a broader, more general category that covers the meanings of all codes. Fitting of the codes into themes was constantly checked and shifted to appropriate themes if in case the codes fitted were not coherent with the theme and codes within the theme. Open codes were categorized into 6 categories of axial codes i.e. causal conditions, context, intervening conditions, central phenomenon, strategies, and consequences as given by Corbin and Strauss[14, 15]. These axial codes were further analyzed to develop selective coding and to generate theory and themes. Separate Excel sheets were prepared for each theme with detailed open codes. The summary was developed in the Microsoft word along with verbatim to justify the theme and include all concepts. Data analysis was performed systematically and rigorously to reflect the views of all participants through constant recheck with the transcript of interviews and audio records.

## Results

**Fig. 1:**
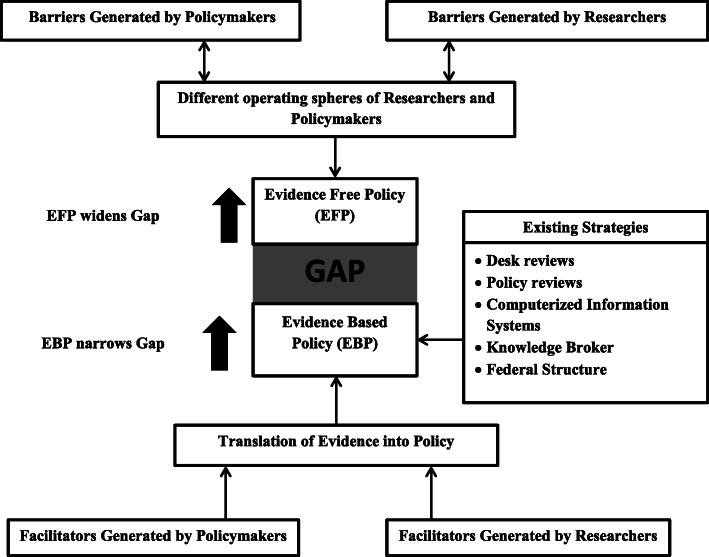
Evidence-based Policymaking in Nepalese Context.

Figure 1 is a schematic representation of identified thematic areas, the interaction between those areas and how it results in EBP in the Nepalese context. Table 1 provides a summarized list of the barriers and facilitators from the sides of both research push and pull.

**Table.1:** Barriers and facilitators identified by Health Policymakers and Researchers

	Barriers	Facilitators
**Health Policymakers**	• Political influence • Poor culture of evidence utilization • Preference of anecdotal evidences • Donor agency’s influence • Poor expenditure for research	• Targets and commitments made at international conferences • Expert/ Stakeholder’s consultation
**Health Researchers**	• Issues of generalizability and credibility of evidences • Poor quality and trust of evidences • Low availability of priority-based researches • Poor targeted dissemination	• Few priority-based researches • Availability of nationally representative surveys • Publication of one-pager research • Partial CE study

As in seen in Fig. 1, these barriers create two different operating spheres for health researchers and policymakers with a fragile connection between them. If these barriers are neglected and more importantly practiced repeatedly, the two different spheres move further apart from each other. As the distance between them increases, we rapidly move towards EFP on the research-to-policy spectrum. To reduce the distance created by these barriers, there exist facilitators generated by both parties and some existing strategies strengthening the links between those two spheres. Thus, in Fig. 1, we can observe a gap between EFP and EBP, whose strength depends on barriers, facilitators, and existing strategies. The barriers widen the gap whereas the facilitators and existing strategies help to narrow it, leading to the formation of EBP, our ultimate goal.

### Perception towards Evidence-Based Policymaking

Under this theme, we discuss the perception of participants on EBP in terms setting national policy agenda, evidence considered during the policy making process and the term policy itself. During the interviews with participants (participant’s profile is provided in Annex 1), it was revealed that three factors played major roles in setting national policy agendas i.e., government and its entity, political influence, and external developmental partners (EDPs). In Nepal, usually the concerned ministry or its department who realizes the need for a certain policy in their line of work, developed policies using the template provided by National Planning Commission (NPC) [16]. When viewed superficially, the process looks fairly systematic and appropriate but in reality, it was either bureaucrats or the EDPs who influenced these government entities behind the scenes.

*“Entity of ministry that works for the issue and takes the responsibility for implementation of policy and its execution will decide and bring out the policy for example if it is related to maternal health, it will be family health division.”* P01.

Similarly, most of the health policymakers highlighted that past policy reviews are often used to set the agenda. They identified evidence such as prevalence studies, data from routine information systems, and various surveys along with the international commitment helped to guide national agendas and decision making.

When looking at the perception of health researchers on policy, most of them perceived it as a form of commitment from the government and reflected through their actions. In addition, researchers perceived that the policies have a role in every step of research, and few even considered policies increased the scopes for research opportunities.

*“Policy I think it defines research. For example, there’s a policy addressing child health but some of its components are not being implemented then, research helps to find out the reasons behind it and ways forward. So looking policy from the research aspect, it provides research opportunities and increases its scope.”* R02.

#### Theme 1: Barriers Generated by Health Policymakers

Under this theme, we discuss the different barriers that are being created through the sides of health policymakers in evidence utilization during policymaking process. As mentioned earlier, political leaders are major influencers of the policy formulation process and ultimately, on how the evidence was to be utilized in the due process. But unfortunately, in recent years, there had been a proliferation of policies with support from policymakers, not as a measure to solve health problems of the country rather, as a means to strengthen their resume and boast about the number of policies developed during their leadership. As a result, these policies did not capture the real need of the population. Moreover, participants highlighted that the relationship between bureaucrats and the vision of the leading political party also influenced the evidence utilization for decision making.

*“In these recent years, there is a race among ministers to implement the maximum number of policies to show it as their success, rather than developing policies based on actual needs. The policy developed in such a way has no implementation and costing plans. We term this trend as “policy ko kheti”* (developing policies as sowing the field).*”* P01.

The impact of that political influence is best described by the common statement used by the participants “Evidence are being utilized but not in the amount it should be”. On top of it, the poor technical competency of policymakers to understand research findings led to a poor culture of scientific evidence utilization and increased preference towards anecdotal evidence.

*“It’s about our practice of preference towards anecdotal evidence, for example, if a certain policymaker had visited Rukum once and had seen a certain situation and the factors behind it, they tend to generalize the context into national level but in reality, it may or may not be true because in another district some other factors might be creating that very situation.”* R02.

These scenarios further demotivated researchers to conduct research on priority areas and due to the lack of such researches, anecdotal evidence were preferred again. Furthermore, donor agencies pushed their agendas to the government through partnerships and implemented novel projects prior to conducting impact studies in the local context leading to less promotion for evidence generation.

*“People get motivated to generate quality data if the generated data is used and vice versa. So, the use of data is one of the barriers to new research. If there is a good practice of utilizing data in decision making, definitely that will drive more research and surveys. Thus, we need to analyze the data availability; where the data gap is, and how to fill the gap.”* R06.

Moreover in Nepal, research is considered a luxurious commodity so, the level of priority placed on it, allocated resources and future growth plans are poor.

*“When talking about evidence, we need to look at our system, how it has been running since past, for example, the plans are prepared at the central level and budget is decided before plans are finalized and as research is not a priority, it gets a very low budget.”* R01.

#### Theme 2: Barriers Generated by Health Researchers

Here, we investigate the barriers that are being created by health researchers that hindering the process of evidence utilization in policymaking process. Most policymakers and even few researchers felt that small-scaled researches are not preferred for evidence utilization as these raise some serious issues of generalizability. Linked to this issue, is the question on the credibility of the evidence and the researchers producing it, for instance, researcher’s skills, vague recommendations, and most importantly researcher’s tendency to highlight results that prove their research objectives while hiding the ones that do not. This leads to the development of mistrust among policymakers towards researchers, making it even more difficult to convince them. As a result, policymakers preferred large-scale surveys conducted by the institutions working closely with them. It is a good practice by policymakers as they trusted quality evidence but this also means that they overlooked other quality findings obtained from researches, both large and small-scale, conducted by organizations or individuals unfamiliar to them.

*“Sometimes quality and trust of evidence also play a factor. For example, Global Burden Disease is calculated by both WHO and IHME but Nepalese policymakers prefer WHO’s data than IHME’s data even though these two organizations use the same methodology because WHO has been working as a partner of government for a long time and policymakers have developed trust towards them.”* R03.

In addition, the poor culture of targeted dissemination by researchers was another barrier to evidence utilization. Every day numerous researches are being conducted at the either organizational or individual level but their potentials are limited in the form of reports collecting dust on shelves, thus leaving policymakers oblivious of the evidence pool.

*“Dissemination happens and researchers try to disseminate in different forums but targeted dissemination is lacking. Also, researchers disseminate findings using technical terms that are difficult for policymakers to understand.”* R07.

#### Theme 3: Different Operating Sphere of Health Policymakers and Researchers

In theme three, we look at how all these above-mentioned barriers resulted in two different spheres of health researchers and policymakers or as termed by a participant *“Two Culture Hypothesis”*. The two culture hypothesis is the concept where researchers and policymakers operate in a different environment with a frail link connecting them i.e. researches being conducted based on the researcher’s self-interest, without consideration of the nation’s priority, and on other hand, to make the condition worse, policymakers formulating national policies based on political influence and anecdotal evidence. This practice further weakened the already frail link between policymakers and researchers leading to the formation of evidence-free policy.

#### Theme 4: Facilitators Generated by Health Policymakers

On a brighter side, in this theme we discuss few facilitators practiced at policymakers’ level to promote evidence-based policymaking. The international commitment was identified by most participants to be a major factor to set the national policy agenda and identify policy areas. Being signatories of these commitments, the government implemented its strategies which may include but are not limited to an increased budget for research, the compulsion to use evidence during policy formulation, and such.

*“Well, these international commitments give us target such as where to reach in-terms of certain issues such as MMR. Also, these provide us some guidelines, for example, due to SDG commitment a minimum of 2 % of the health budget is allocated for research and due to partnership with WHO, the Nepal government needs to gradually increase the budget in the health sector to 10 %.”* P05.

Similarly, few Nepalese researchers and policymakers identified experts/ stakeholder’s consultation as one of the factors that promoted evidence utilization.

#### Theme 5: Facilitators Generated by Health Researchers

Similarly, in this theme, we discuss the different facilitators practiced by health researchers to promote EBP. Most policymakers, as well as researchers, identified that the researches that are done based on the government’s priority and with the involvement of policymakers increased the chance for the evidence to be utilized in the policymaking process.

*“Lawrence Green once said for evidence-based practice we need practice-based evidence thus, for the evidence-based policy we first need a practice of policy-based evidence. We think that if we present research evidence, it should guide policy but in reality, it does not occur. It is difficult to go that route from the beginning so first, we need to practice research to be guided by policy and policy priority identified by government and gradually move towards guiding policy by research evidence.”* R03.

These priority areas are identified by holding close discussions between researchers and policymakers. Furthermore, this nature of collaboration increased ownership among policymakers.

*“Those researches conducted under guidance from the ministry are easy to translate into policy but those conducted at an individual or organizational level are hard to translate and is time consuming mainly due to the issues of trusting the finding from the research.”* R01.

These sorts of collaboration are observed in nationally representative surveys such as Nepal Demographic and Health Survey (NDHS), STEPs, and Multiple Cluster Indicator Surveys (MICS) and thus, policymakers are aware of it and most likely utilized it during policy development.

In the previous section, lack of targeted dissemination was identified as a barrier, thus an appropriate communication approach such as one-pager policy brief in the Nepali language solved the issue. These briefs provided a short description of the findings and specific recommendations with minimal use of technical terms. This initiative helped policymakers understand the evidence and utilized them during policy development.

*“One-pager research brief will benefit as well because no policymaker has time to read 40-50 pages report. So one-pager with key findings and recommendations will help them understand the situation and will be easier to discuss as well.”* R02.

Moreover, there had been few instances in Nepal where findings from cost-effectiveness studies for certain programs are utilized. These effectiveness studies gave insights on the feasibility of the programs and implementation module.

*“While developing nursing and midwifery policy, we decided to have a nurse in each ward and thus performed cost analysis where we found that cost for producing a single ANM is about 5-6 lakhs and we have about 300 wards so the cost came huge and on top of that we also need to provide the salary. Another example is of placing school health nurse, there are 36,000 schools in Nepal, and the basic salary of staff nurse is 23,500 so the cost for the single year came to about 9 Arba, which we cannot bear every year thus, in these cases we decided to go phase-wise based on data from costing.”* P04.

#### Theme 6: Existing Strategies to promote Evidence-based Policymaking

Apart from the health researcher’s and policymaker’s practices to promote EBP, in this theme we discuss the different strategies that are in place within the national health care system of Nepal that promotes EBP. First one is the desk review as a compulsory step during policy development, which helped to reduce the gap between evidence not being utilized and their utilization.

*“The trend of desk reviews is prevalent in the health sector. For example, if the policy is going to be developed on certain themes, then available literature is collected through desk reviews. We do not have a specific guideline for a literature review so we look for published and unpublished researches in Nepalese context that are available to us.”* P01.

Many policymakers identified policy reviews to be one of the factors used to determine the country’s priority goals. These policy reviews, though often irregular in practice, gave priority areas and there had been instances of researches, large and small-scale, being conducted based on these priorities.

*“While developing health policies, it’s not like that we don’t have any policies till date, we do have some which are reviewed may be in 5 years, 7 years or 10 years and based on review finding we either update the existing policy or develop new policy. Similarly, we come across new policy issues during the yearly review as well.”* P03.

At the time of data collection, the concept of evidence facilitators or *“knowledge broker”*, a term used by a participant, was rising. A knowledge broker may be an institution or an employee of a certain institution whose job is to make current evidence available at the policymaker’s table. Because of these knowledge brokers, the policymakers easily got their hands on quality and updated information to make evidence-based decisions.

The practice of regular updates and uptake of the computerized routine information system in Nepal, recently District Health Information System 2 (DHIS 2), helped the very ground level health facility to get quality information in real-time. This ease in accessibility of quality information enabled local government to plan programs accordingly. Moreover, the implementation of a new federal structure provided an opportunity for local government to exercise more power to plan programs based on their context and quality information provided by the routine information system.

#### Theme 7: Recommendation for better Evidence-based Policymaking

Along with identifying the barriers, facilitators and strategies in place to promote EBP in Nepal, the study also focused on different approaches that can be taken by both health policymakers and researchers, identified from themselves, for its better practice. These suggested actions are presented in this theme in Table 2 below.

**Table.2:** Recommendations to Health Policymakers and Researchers for better EBP

Recommendations to Health Policymakers	Recommendations to Health Researchers
• Promote the research practice by utilizing them. It motivates researchers to conduct more studies.	• Actively push their research findings and recommendations to policymakers as they do not have time to search for evidence during decision making.
• Identify priority areas on which researchers can conduct researches and help the EBP process.	• Conduct studies on identified priority areas by the government so the policymakers can utilize those researches to promote the practice of EBP.
• Establishment of a formal forum of health policymakers and researchers with priority on evidence sharing to improve the fragile link between them.	• Involve policymakers since the beginning of their research to develop ownership among them, resulting in the use of generated evidence to develop appropriate actions.
• Promotion of impact study before the policy and program implementation to promote the research practice and evidence utilization; further motivating researchers to conduct more research.	• Publication of research briefs along with recommendations for policy, in plain language preferably in Nepali.

## Discussion

Studies looking at EBP have identified a variety of strategies used by policymakers to set national policy agendas. In this study, the government and its entity, political influence, and EDPs were identified as major influential factors for setting such agendas. A similar study conducted in Nepal [4] identified political influence, research evidence, and personal experience of policymakers as influencing factors whereas, a study in Argentina [6] identified cost to the state, meeting donor requirements and self-interest of policymakers. Setting national policy agendas is a complex task and many factors come into play when determining it. We can observe in both a lower-middle-income country [17] like Nepal and in an upper-middle-income country [17] like Argentina, political influence, and EDPs play major roles in this process.

Through this study, we found that though sometime evidence are utilized, political agendas triumph over it with the only point of consolation being the utilization of evidence, to some extent, in support of that decision. However, in recent years we have seen a positive move towards EBP where policymakers have been giving priority for some national-level researches and their utilization. Unfortunately in Nepal, this trend tends to be limited to the inclusion of evidence as a statement in the background of the policies rather than being utilized to guide the strategies and activities of the policies.

Barriers that were identified by Nepalese policymakers and researchers which also appeared in other published studies include political influence during decision making, resource constraints, a poor culture of evidence utilization, issues of generalizability and credibility of research, quality and trust of evidence being available to health policymakers, poorly targeted dissemination, low availability of priority-based research, use of jargons and technical competencies of policymakers [3, 4, 6–11, 18–20]. Other barriers identified in the study include the preference of anecdotal evidence during policy formulation and the influence of EDPs.

Due to the lack of targeted dissemination, the evidence is not reaching the tables of the policymakers and moreover, the less use of evidence demotivates researchers to conduct more studies. As a researcher, we are eager to blame policymakers for not utilizing evidence but we are also not fulfilling our responsibility of disseminating findings. The scale and quality of research primarily focus on generalizability, credibility, trust, validity, and reliability of the research. As most of the small scale researches are done at the local level with few samples, it raises the question on the quality of information generated. Thus, policymakers who make national-level policies do not prefer them. Moreover, it is extremely difficult to convince policymakers to trust research findings generated by sources unfamiliar to them. This might either be because they were not involved in research or due to their poor knowledge regarding research terms such as p values. As a result, policymakers and researchers operate on two different spheres and fail to not perceive each other’s views on the research. For example, we have little to no priority-based evidence, mostly because researches are conducted based on the researcher’s self-interest and also due to lack of promotion of priority areas by policymakers. Policymakers perceive research to be a luxurious commodity and researchers perceive policymakers to be unknown about research concepts thus creating a gap for evidence utilization. Further increasing the already existing gap is the resource constraint where the budget allocated for research is very low. The combination of all these factors has led to the formulation of policies that utilize little to no scientific evidence.

Similar to barriers, facilitators to EBP identified by Nepalese policymakers and researchers that also appeared in other published studies include stakeholders and/or expert consultation, conduction of research on nationally identified priority areas, the culture of targeted dissemination, the practice of cost-effectiveness study, and involvement of policymakers during the research [4, 6, 8, 9, 11, 19–21]. Unique to Nepal at the time of data collection include targets and commitments made as a part of signatory country during any conference or such, availability of few nationally representative surveys, and publication of a one-pager research brief with clear policy recommendations. Similarly, Nepalese policymakers and researchers mentioned strategies that are in place or mandatory during policy formulation that is promoting EBP in the Nepalese context. These strategies include mandatory desk reviews, the practice of policy reviews, regular update and uptake of the computerized routine information system, promotion of the evidence facilitators, and the country’s move towards federal government structure allowing more autonomy to local government to identify problem areas, conduct research and implement local policies/ activities accordingly.

As mentioned before, not every policy is based on anecdotal evidence; scientific evidence is also utilized mostly due to the mandatory desk reviews. But the utilization of current and relevant evidence during policy formulation is still a question. As stated by researchers, factors such as large scale nationally representative surveys and the involvement of policymakers during the research help for evidence to get utilized. Similarly, policymakers stated exchange efforts such as targeted dissemination and publication of a one-pager research brief can help policymakers be aware of the findings. Moreover, the development of mass communication technologies especially the increasing use of social media and promotion of findings through it helps to reach policymakers easily. Similarly, the promotion of evidence facilitators in recent times has helped to reduce the gap between research and policy. A new computerized routine information system provides a new opportunity for research evidence to be utilized more at the local level in the new federal structure.

Comparing the recommendation provided by Nepalese policymakers and researchers for better EBP with the recommendation mentioned in different published journals, communication is identified as a major tool [4, 7, 9, 21]. To improve the communication channel between policymakers and researchers, both of these parties need to take action. Policymakers can establish a formal forum where both of them can meet together and share findings whereas, researchers can work for better-targeted dissemination of research findings and recommendations in the language policymakers can understand and utilize. Apart from these, Nepalese policymakers and researchers identified promotion for the practice of impact study prior to project/ policy implementation and promotion of systematic reviews and meta-analysis on the nation’s priority areas for better EBP. Through this, we can see that the problem of EBP is similar in the global context and needs similar actions to be taken here in our national context so that the policies that are now formed will be based on evidence and address the actual needs of the people.

## Limitations

The study was based on the sample of researchers and policymakers who were interested in participating and willing to speak with us. Thus, the sample is limited by the non-random selection of participants. Similarly, the study was an academic research project for obtaining an undergraduate degree, thus allocated time of one month for data collection was a constraint and academic researchers were not included as research participants. In addition, due to the time constraint, during the data collection repeat interviews could not be conducted, the transcripts were not returned to participant for comment as well as they were not consulted to provide feedback on the findings.

## Conclusions

This research is unique in its focus on both the demand and the supply side of evidence utilization conducted in the context of a lower middle-income country like Nepal. It explores how both sides perceive each other and identifies key barriers and facilitators to EBP along with potential measures to lessen the gap that exists between them. It was identified that though the research evidence and policy analysis documents were considered in the policymaking process, political influence played a major role in setting national policy agendas. The research sheds light on importance of research development along with its utilization culture to minimize the gap that exists between policymakers and researchers for implementation of policies. For this, better communication strategies can act as a major potential measure for a comprehensive and united vision of policymakers and researchers to promote EBP. These strategies include the establishment of a formal forum of policymakers and researchers, better-targeted dissemination of findings, and identification of the nation’s priority areas for research. Apart from these strategies, promotion of practice for impact study prior to policy implementation and promotion of systematic reviews and meta-analysis could further help for better EBP.

## Data Availability

The data underlying this article cannot be shared publicly to maintain the privacy of individuals that participated in the study. The data will be shared on a reasonable request to the corresponding author.

## Abbreviations

BPH: Bachelor’s in public health
DHIS 2: District Health Information System 2
DoHS: Department of Health Services
EBP: Evidence-Based Policymaking
EDPs: External Development Partners
EFP: Evidence-Free Policymaking
IHME: Institute for Health Metrics and Evaluation
MICS: Multiple Indicator Cluster Surveys
MMR: Maternal Mortality Ratio
MoHP: Ministry of Health and Population
NDHS: Nepal Demographic and Health Survey
NPC: National Planning Commission
PBE: Policy-Based Evidence
SDG: Sustainable Development Goals
WHO: World Health Organization
